# Insights and decision-making on surgical triggers of glaucoma-related complications in congenital cataract surgery: a clinical review

**DOI:** 10.3389/fmed.2025.1622410

**Published:** 2025-09-26

**Authors:** Bin Lin, Wei Fan, Dong-kan Li

**Affiliations:** ^1^Xiamen Eye Center and Eye Institute of Xiamen University, School of Medicine, Xiamen, China; ^2^Xiamen Clinical Research Center for Eye Diseases, Xiamen, Fujian, China; ^3^Xiamen Key Laboratory of Ophthalmology, Xiamen, Fujian, China; ^4^Fujian Key Laboratory of Corneal & Ocular Surface Diseases, Xiamen, Fujian, China; ^5^Xiamen Key Laboratory of Corneal & Ocular Surface Diseases, Xiamen, Fujian, China; ^6^Translational Medicine Institute of Xiamen Eye Center of Xiamen University, Xiamen, Fujian, China

**Keywords:** congenital cataract, postoperative glaucoma, surgical factors, surgical methods, surgical timing, medication treatment

## Abstract

Glaucoma-related adverse events (GRAE) after congenital cataract surgery severely affect the visual recovery of children and have attracted significant attention in the medical community. This article focuses on the research of its surgical factors. In terms of age, the younger the age at surgery, the higher the risk of glaucoma-related adverse events after surgery. Regarding surgical methods, primary in-the-bag intraocular lens (IOL) implantation, secondary in-the-bag IOL implantation, and ciliary sulcus IOL implantation have different effects on the incidence of glaucoma. There are controversies over the advantages and disadvantages of different implantation methods and the definition of high-risk factors. In terms of medications, corticosteroids used to control inflammation may induce elevated intraocular pressure, and the safety data of intraocular pressure-lowering medications in children are incomplete. To balance the contradictions among surgical methods, surgical timing, and medication use, it is necessary to closely monitor the intraocular pressure, anterior segment structure, and the space of the posterior segment during the perioperative period. When the intraocular pressure rises, the cause should be identified clearly and targeted treatment should be carried out. When using medications to lower intraocular pressure, drugs with fewer adverse reactions in children should be preferred. Minimally invasive glaucoma surgery (MIGS) is a promising option for refractory cases. Further research is needed in the future to clarify the risk factors, optimize treatment strategies, reduce the incidence of glaucoma-related adverse events after congenital cataract surgery, and improve the visual prognosis of children.

## 1 What is glaucoma-related adverse events (GRAE) after congenital cataract surgery?

Congenital cataract is a major threat to the visual development of infants and young children. In recent years, advancements in cataract surgery techniques have enabled medical professionals to address this issue by making the refractive media of the eyes transparent again through cataract surgery (1). Meanwhile, intraocular lens implantation has become the standard method for correcting refraction after lens extraction (2).

However, the process of restoring normal vision in children with congenital cataracts is accompanied by multiple complex clinical challenges. Deciding when to implant an intraocular lens after cataract extraction is not straightforward. It requires a comprehensive assessment of multiple factors, including the age of the child (3, 4), the overall development of the eyes (5, 6), and the specific characteristics of the cataract. Form-deprivation amblyopia often accompanies congenital cataracts, and its management also presents a significant clinical challenge (7). Since children with congenital cataracts often have other complex eye problems, perioperative management faces challenges in achieving favorable clinical outcomes (8).

Among them, in the prevention of postoperative complications, transient elevation of intraocular pressure and the development of secondary glaucoma are common problems. These conditions can lead to irreversible damage to the optic nerve and a significant decline in visual function. In particular, GRAE after congenital cataract surgery have become a particularly serious issue and have received increasing attention from the medical community (9). Studies have shown that glaucoma-related adverse events are likely to occur after pediatric cataract surgery. Most cases are diagnosed within 2 years after surgery, with an early-onset rate of 33.7%, and the reoperation rate after trabeculectomy is as high as 50% (10).

Given that it may cause severe and permanent visual impairment, there is an urgent need for further research and innovative therapeutic strategies to effectively manage this complication. Since the congenital factors of patients are uncontrollable, while surgical-related factors are controllable, this article aims to conduct a study on the surgical-related factors involved in the occurrence and development of GRAE after congenital cataract surgery. This clinically oriented review can assist clinicians and scholars in judging and reducing the incidence of postoperative GRAE.

## 2 Methods

### 2.1 Literature search strategy

To comprehensively synthesize the surgical triggers of glaucoma-related adverse events after congenital cataract surgery and expand the discussion scope of this review, we employed a hybrid approach that combines systematic literature retrieval (based on the aforementioned database-specific strategies) with flexible expansion—supplementing with relevant literature appropriately according to the content of the finally screened studies. This approach aims to reduce the limitations of relying solely on retrieval strategies and ensure both the rigor of the review and the breadth of its discussion.

**Figure 1:**
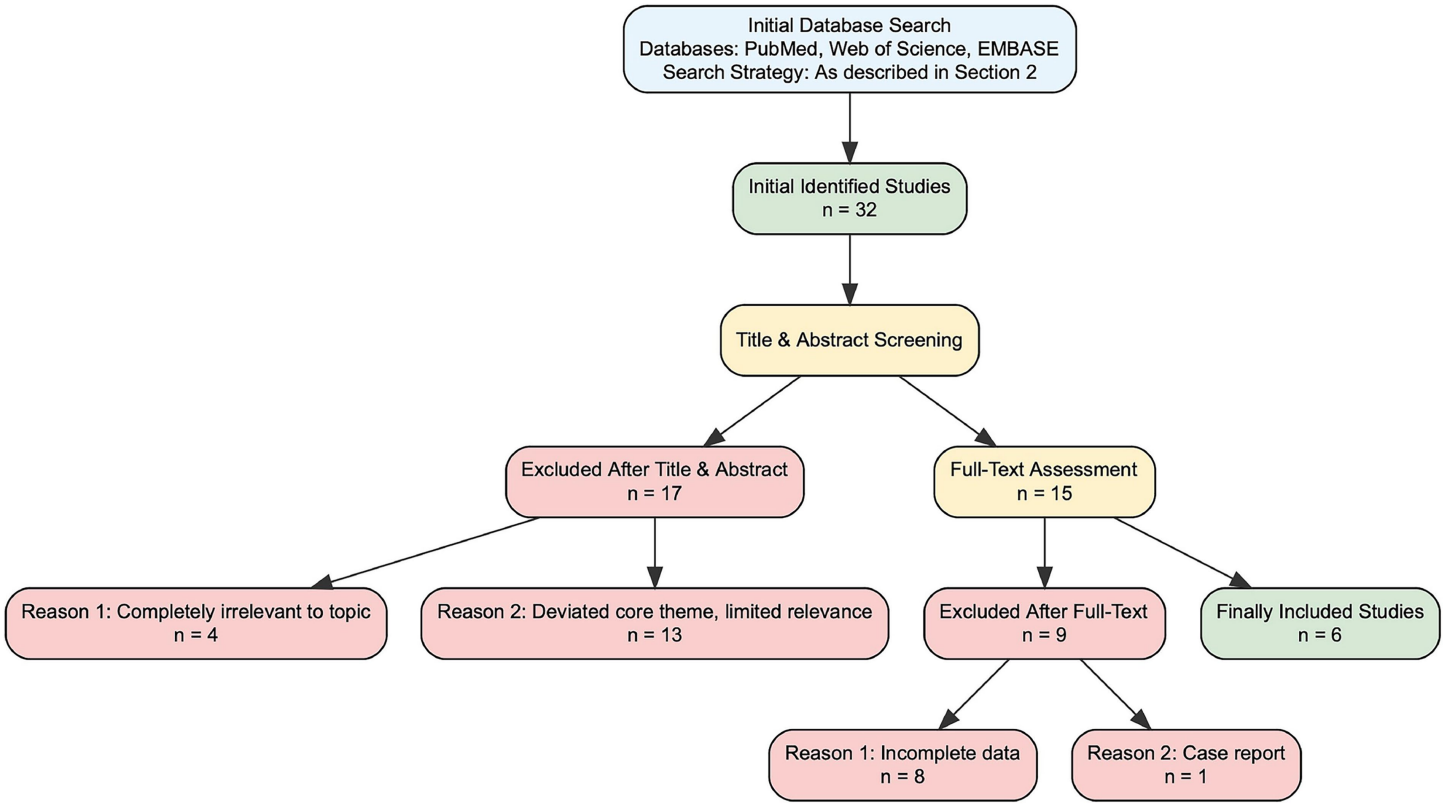
Flow diagram of literature screening process. This flow diagram illustrates the stepwise screening process of literature included in this review, in accordance with the PRISMA (Preferred Reporting Items for Systematic Reviews and Meta-Analyses) guidelines. The initial search across PubMed, Web of Science, and EMBASE databases identified 32 studies. After title and abstract screening, 17 studies were excluded (4 for complete irrelevance to the review topic, 13 for deviated core themes with limited relevance). The remaining 15 studies underwent full-text assessment, during which 9 additional studies were excluded (8 for incomplete data, 1 for being a case report with a sample size ≤5). Finally, 6 studies met all inclusion criteria and were included in the review for data synthesis and analysis.

This review systematically searched three core biomedical databases to ensure comprehensive coverage of relevant literature. The search period spanned from the inception of each database to Apr 1, 2025. A combination of Medical Subject Headings (MeSH terms) (for PubMed), Emtree terms (for EMBASE), and free-text words was used, with consistent Boolean logic applied across databases to target research on surgical triggers of glaucoma-related adverse events (GRAE) after congenital cataract surgery. The specific Retrieval Strategies and literature inclusion/exclusion criteria are as follows. The detailed literature screening flowchart is presented in Figure 1.

#### 2.1.1 Database-specific retrieval strategies

##### 2.1.1.1 Database retrieval strategy

###### 2.1.1.1.1 PubMed

(“Congenital Cataract”[MeSH Terms] OR “Infantile Cataract”[Text Word] OR “Pediatric Cataract”[Text Word])

AND

(“Cataract Extraction”[MeSH Terms] OR “Cataract Surgery”[Text Word] OR “Intraocular Lens Implantation”[MeSH Terms] OR “IOL Implantation”[Text Word] OR “Primary IOL Implantation”[Text Word] OR “Secondary IOL Implantation”[Text Word] OR “Ciliary Sulcus IOL Implantation”[Text Word])

AND

(“Glaucoma”[MeSH Terms] OR “Glaucoma-Related Adverse Events”[Text Word] OR “Intraocular Pressure Elevation”[Text Word] OR “Secondary Glaucoma”[MeSH Terms] OR “Postoperative Glaucoma”[Text Word] OR “Pediatric Glaucoma”[Text Word])

AND

(“Surgical Factors”[Text Word] OR “Surgical Timing”[Text Word] OR “Corticosteroids”[MeSH Terms] OR “Intraocular Pressure-Lowering Drugs”[Text Word] OR “Risk Factors”[MeSH Terms] OR “Trabeculectomy”[MeSH Terms] OR “Minimally Invasive Glaucoma Surgery”[Text Word] OR “MIGS”[Text Word])

###### 2.1.1.1.2 Web of Science (WoS)

TS = (“congenital cataract” OR “infantile cataract” OR “pediatric cataract”).

AND

TS = (“cataract extraction” OR “cataract surgery” OR “intraocular lens implantation” OR “IOL implantation” OR “primary IOL implantation” OR “secondary IOL implantation” OR “ciliary sulcus IOL implantation”).

AND

TS = (“glaucoma” OR “glaucoma-related adverse events” OR “intraocular pressure elevation” OR “secondary glaucoma” OR “postoperative glaucoma” OR “pediatric glaucoma”).

AND

TS = (“surgical factors” OR “surgical timing” OR “corticosteroids” OR “intraocular pressure-lowering drugs” OR “risk factors” OR “trabeculectomy” OR “minimally invasive glaucoma surgery” OR “MIGS”).

###### 2.1.1.1.3 EMBASE

(“congenital cataract”/exp. OR “infantile cataract” OR “pediatric cataract”)

AND

(“cataract extraction”/exp. OR “cataract surgery” OR “intraocular lens implantation”/exp. OR “IOL implantation” OR “primary IOL implantation” OR “secondary IOL implantation” OR “ciliary sulcus IOL implantation”)

AND

(“glaucoma”/exp. OR “glaucoma-related adverse events” OR “intraocular pressure elevation”/exp. OR “secondary glaucoma”/exp. OR “postoperative glaucoma” OR “pediatric glaucoma”/exp)

AND

(“surgical factor” OR “surgical timing” OR “corticosteroid”/exp. OR “intraocular pressure-lowering drug” OR “risk factor”/exp. OR “trabeculectomy”/exp. OR “minimally invasive glaucoma surgery” OR “MIGS”)

### 2.2 Literature inclusion criteria

#### 2.2.1 Type

Original research (RCTs, cohort studies, case–control studies), systematic reviews, meta-analyses, or clinical observations focusing on congenital cataract surgery and postoperative GRAE; reviews with clear retrieval and analytical logic were also included.

#### 2.2.2 Population

Pediatric patients (≤18 years) with congenital/infantile cataracts who underwent cataract surgery (primary/secondary IOL implantation, ciliary sulcus IOL implantation, etc.).

#### 2.2.3 Outcomes

Studies reporting surgical factors (age, methods), medication factors (corticosteroids, intraocular pressure-lowering drugs), postoperative GRAE incidence, or GRAE mechanisms.

#### 2.2.4 Availability

Full-text articles in English with complete data (excluding abstracts, conference proceedings, or studies with incomplete outcomes).

### 2.3 Literature exclusion criteria

#### 2.3.1 Irrelevant topics

Focus on acquired cataracts (e.g., age-related, traumatic) or glaucoma unrelated to congenital cataract surgery (e.g., primary congenital glaucoma).

#### 2.3.2 Insufficient data

Unclear design, incomplete outcomes (e.g., unreported surgical timing), or unextractable key information (e.g., unknown IOL type).

#### 2.3.3 Low evidence level

Case reports (sample size ≤5), animal/*in vitro* studies, or expert opinions without systematic literature support.

#### 2.3.4 Duplication

Duplicate publications (latest/comprehensive version selected).

## 3 What factors are mainly related to the risk of postoperative glaucoma-related adverse events?

### 3.1 Age factor

The risk of postoperative GRAE is significantly elevated in children with congenital cataracts who undergo cataract surgery at 1–6 months of age (11). Some studies have suggested that among infants with unilateral congenital cataracts, the incidence of glaucoma 5 years after surgery is 26% in children whose surgical age is ≤ 48 days, while it is 9% in the 49-210-day group (12). Although early surgery may be associated with missed diagnosis secondary to inadequate intraocular pressure monitoring, the younger the age, the poorer the follow-up compliance, resulting in more significant risk accumulation (13). Overall, the risk of glaucoma after congenital cataract surgery is negatively correlated with surgical age. The younger the age, the higher the risk. It is necessary to balance the visual benefits of early surgery against the risk of glaucoma. However, current literature has not yet reached a consensus on the optimal timing of congenital cataract surgery, nor has a unified minimum age limit for the procedure been established.

### 3.2 Surgical plan

#### 3.2.1 Primary in-the-bag intraocular lens (IOL) implantation

Some experts have proposed that early implantation of an IOL has a long-term positive impact on the visual development of children (14). However, it was previously believed that performing cataract extraction and IOL implantation simultaneously in young children might increase the risks of inflammatory reactions and iris synechiae (3, 15). Residual lens matter during surgery or improper handling of the posterior capsule may induce secondary glaucoma. Research shows that using the technique of “continuous circular posterior capsulorhexis + anterior vitrectomy” can reduce the incidence of posterior capsule opacification and glaucoma. It should be noted that residual vitreous during surgery may block the trabecular meshwork. However, some scholars have also found that this surgical combination may cause displacement of the IOL (16). At the same time, the proportion of infants and young children meeting the “ideal conditions” for primary implantation is not high (17), and criteria for defining high-risk conditions (e.g., microphthalmia, microcornea) remain unstandardized.

#### 3.2.2 Secondary in-the-bag IOL implantation

**Figure 2:**
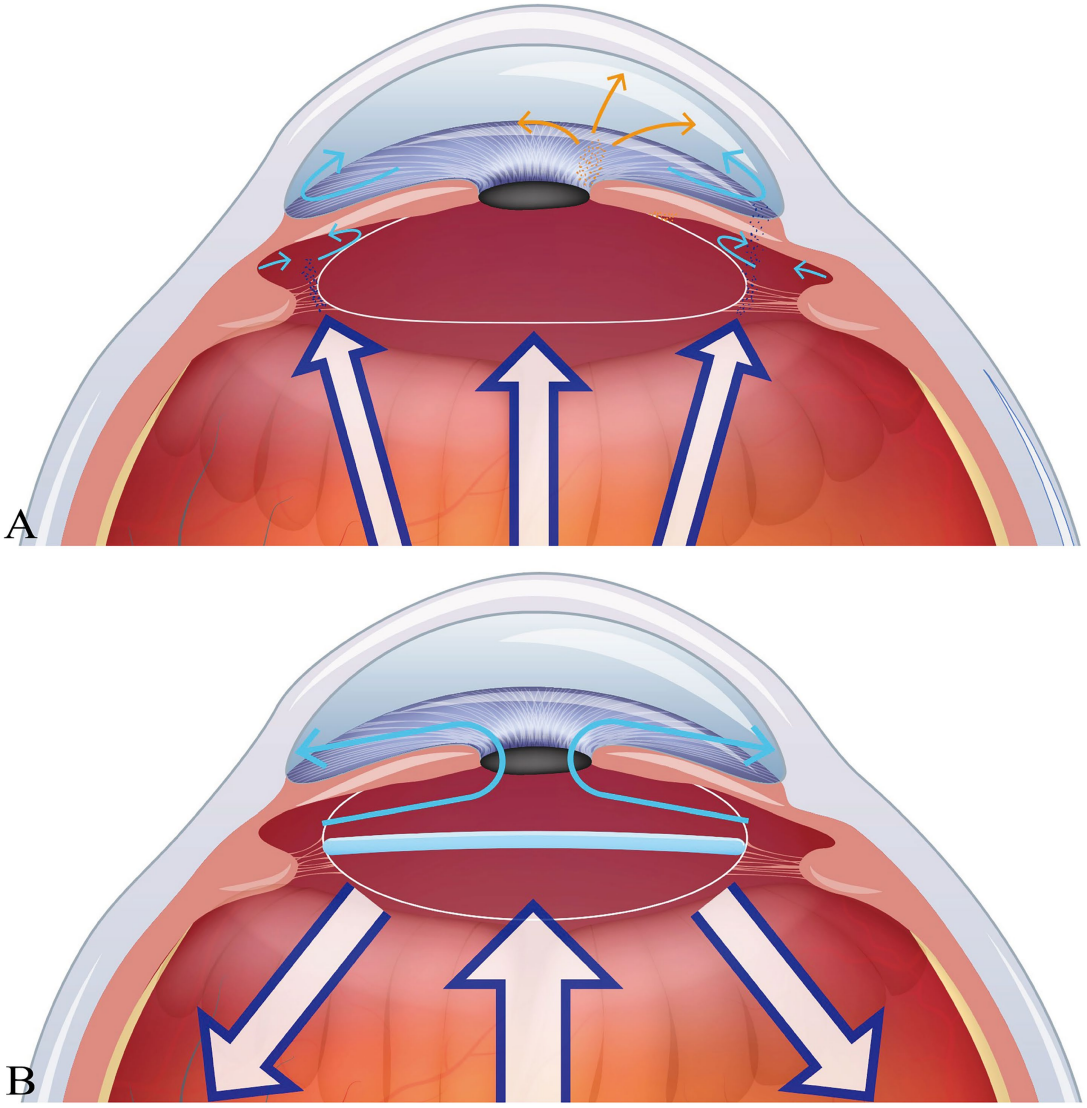
Comparison of the anterior segment conditions in children with congenital cataracts with and without IOL implantation. Part **(A)** shows where the IOL is not implanted primarily after cataract extraction. Due to the lack of support from the IOL, under the pressure of the posterior segment of the eye, the lens capsule and iris tissues experience varying degrees of friction and synechiae, resulting in partial pigment loss. This is both a cause and a consequence of anterior segment inflammation. Secondly, posterior synechiae at the pupillary margin obstructs the aqueous humor circulation, increases the pressure in the posterior chamber, and further narrows or even closes the anterior chamber angle. Some components in the vitreous can more easily stimulate the occurrence of anterior segment inflammation without obstruction. Part **(B)**, on the other hand, shows the situation where the intraocular lens is implanted primarily after cataract extraction, forming a good anterior chamber and posterior chamber structure. It also blocks the disturbance of the anterior segment by the vitreous.

Some studies have shown that children with congenital cataracts can achieve better visual development by undergoing secondary IOL implantation after 2 years old, following early cataract extraction (18). However, previous studies by some scholars have found that the incidence of glaucoma in children who did not receive primary IOL implantation after cataract surgery is significantly higher than that in children who underwent cataract surgery combined with primary IOL implantation (19). The following mechanisms may contribute to a higher incidence of glaucoma in children with secondary IOL implantation. Firstly, IOL implantation can provide mechanical support to the lens capsule, reducing the risk of angle distortion or trabecular meshwork collapse. In the aphakic state, the forward movement of the vitreous may compress the anterior chamber angle or cause chemical damage (20, 21). Secondly, one of the major mechanisms for the occurrence of glaucoma-related adverse events after congenital cataract surgery is the risk of angle synechiae and fibrosis caused by inflammation (22). Primary IOL implantation can reduce the synechiae and friction between different tissues, which may play a positive role in reducing postoperative inflammation. In contrast, the aphakic state may exacerbate inflammation due to residual lens matter or vitreous disturbance. Finally, during infancy, especially in children ≤ 6 weeks old undergoing cataract surgery, the anterior chamber angle is not fully developed. The aphakic state may interfere with the normal formation of the aqueous humor outflow pathway. IOL implantation may reduce this impact by stabilizing the intraocular structure (20). The specific mechanism is shown in Figure 2.

While primary in-the-bag IOL implantation is widely recognized for mitigating postoperative GRAE risk -by stabilizing intraocular structure, reducing angle distortion, and minimizing aphakic-related inflammation (5, 14–16, 20) -secondary in-the-bag IOL implantation retains critical clinical value for pediatric patients, particularly when balancing surgical timing with long-term visual outcomes.

For children with congenital cataracts, ocular growth (e.g., axial length, corneal curvature) is highly dynamic in the first 2 years of life (18). Early primary IOL implantation, although beneficial for reducing GRAE, may be limited by the unpredictability of pediatric ocular development. The IOL power calculated based on infantile ocular parameters often becomes mismatched as the child grows, leading to refractive errors (e.g., myopic shift) that hinder visual development. In contrast, secondary IOL implantation -typically performed after 18–24 months of age, when ocular growth slows and parameters stabilize (18) -enables more accurate IOL power calculation. A study of pediatric patients with congenital cataracts (including those with complex ocular phenotypes) showed that secondary implantation reduced refractive error-related visual delays. The mean absolute refractive error at 5-year follow-up was 0.75 ± 0.32 D in the secondary implantation group, significantly lower than 1.23 ± 0.45 D in the primary implantation group. This precision is critical for optimizing retinal image quality, a key factor in preventing amblyopia and promoting normal visual pathway development in children.

Additionally, secondary implantation allows for a more thorough assessment of the child’s ocular condition before IOL placement. For example, in cases where postoperative inflammation persists or anterior chamber angle development is incomplete after initial cataract extraction, delaying IOL implantation until these issues resolve reduces the risk of IOL-related complications (e.g., iris synechiae, chronic uveitis) that could indirectly exacerbate GRAE. This “wait-and-assess” approach aligns to minimize both GRAE risk and visual development barriers, as stable ocular conditions at the time of secondary implantation ensure better IOL positioning and long-term biocompatibility.

Notably, the advantage of secondary implantation in optical precision does not negate the GRAE risk-reduction benefit of primary implantation. Instead, it highlights that surgical decision-making must prioritize individual pediatric patients’ needs primary implantation may be preferred for infants at high GRAE risk (e.g., those with a family history of glaucoma), while secondary implantation is more suitable for children where optimizing refractive accuracy and visual development takes precedence (18, 20).

#### 3.2.3 Ciliary sulcus IOL implantation

Due to the complex ocular conditions of children, when posterior capsule complications occur during cataract surgery or capsular bag opening is difficult during secondary IOL implantation, surgeons often implant the IOL in the ciliary sulcus. Some studies have found that IOL implantation in the ciliary sulcus has a significant impact on glaucoma (23). Multivariate analysis shows it is an independent risk factor for secondary glaucoma (OR 1.39, 95% CI 1.07–4.85, *p* = 0.04). Seven out of every nine patients with secondary glaucoma have the IOL implanted in the ciliary sulcus, indicating a significant increase in risk. From the perspective of ocular structure, IOL implantation in the ciliary sulcus is likely to cause multiple abnormalities. It increases the probability of iris friction, posterior synechiae, and chronic uveitis (24). Although the internal mechanism leading to secondary glaucoma is unclear, the increased risk is a fact. The IOL located in the ciliary sulcus can also cause changes in the anterior chamber angle structure. It may narrow the anterior chamber angle, alter the aqueous humor outflow path, and affect the intraocular pressure. When the normal outflow of aqueous humor is blocked, the intraocular pressure rises, further increasing the risk of glaucoma (12, 25), which is similar to the theory mentioned above.

### 3.3 Medication factors

As mentioned above, the occurrence of GRAE after congenital cataract surgery is associated with intraocular inflammatory reactions. Therefore, corticosteroids are often used to control inflammatory reactions during the postoperative treatment process (26, 27). However, this practice also carries the risk of inducing elevated intraocular pressure (28). Studies on adults have shown that individuals with high corticosteroid sensitivity and those with abnormal trabecular meshwork function are more susceptible to corticosteroid-induced elevated intraocular pressure (29). In infants and children, corticosteroids can cause elevated intraocular pressure more rapidly and with a lower dosage requirement (30).

When elevated intraocular pressure occurs after congenital cataract surgery, drug therapy to lower the intraocular pressure is the primary treatment method. It should be noted that currently, the safety data of most intraocular pressure-lowering medications in infants and children are incomplete. If the medication is not selected appropriately, it will be difficult to achieve effective intraocular pressure control, and it may also cause numerous adverse reactions.

To systematically synthesize the key findings on factors influencing postoperative GRAE in children with congenital cataracts (including age, surgical approaches, and medications), along with their respective risks, mechanisms, advantages/disadvantages, and supporting evidence, Table 1 provides a concise comparative overview for quick reference.

**Table 1:** Summary of factors associated with postoperative GRAE in congenital cataract surgery.

Related factor	Risk of postoperative GRAE	Proposed mechanisms	Advantages	Disadvantages	References
Age factor surgical age	Significantly elevated in 1–6 months old; negatively correlated with surgical age. * ≤ 48 days: 26% glaucoma incidence at 5 years post-surgery. *49–210 days: 9% glaucoma incidence at 5 years post-surgery.	*Younger age associated with inadequate intraocular pressure monitoring, leading to missed diagnosis. *Poorer follow-up compliance in younger children, resulting in risk accumulation. *Underdeveloped anterior chamber angle in infants (especially ≤6 weeks old) may be more vulnerable to surgical interference.	Early surgery may prevent form-deprivation amblyopia and promote visual development.	Early surgery may be associated with a higher risk of GRAE, and no consensus on optimal minimum surgical age or balance between amblyopia prevention and GRAE avoidance.	(3, 11–13, 20)
Surgical approach primary in-the-bag IOL implantation	Moderate; lower than secondary in-the-bag IOL implantation in some studies but associated with residual lens matter/vitreous-related risks.	*Residual lens matter or improper posterior capsule handling induces secondary glaucoma. *Residual vitreous may block trabecular meshwork. *Provides mechanical support to lens capsule, reducing angle distortion/trabecular meshwork collapse; reduces tissue synechiae and inflammation.	*Long-term positive impact on children’s visual development. *Reduces risk of angle distortion, trabecular meshwork collapse, and tissue synechiae. *“Continuous circular posterior capsulorhexis + anterior vitrectomy” reduces posterior capsule opacification and glaucoma incidence.	*May increase risks of inflammatory reactions and iris synechiae in young children. *“Continuous circular posterior capsulorhexis + anterior vitrectomy” may cause IOL displacement. *Few infants/young children meet “ideal conditions” for implantation.	(5, 14–16, 20)
Surgical approach secondary in-the-bag IOL implantation	Higher than primary in-the-bag IOL implantation.	*Aphakic state leads to forward vitreous movement, compressing anterior chamber angle or causing chemical damage. *Exacerbated inflammation due to residual lens matter or vitreous disturbance in aphakic state. *Aphakic state interferes with normal aqueous humor outflow pathway development (especially in infants ≤6 weeks old).	Enables better visual development when performed after 2 years old following early cataract extraction.	*Significantly higher glaucoma incidence than primary in-the-bag IOL implantation. *Lack of IOL support in aphakic period increases risks of angle compression, inflammation, and abnormal aqueous humor outflow pathway development.	(2, 9, 19, 20)
Surgical approach ciliary sulcus IOL implantation	High; identified as an independent risk factor for secondary glaucoma.	*Increases iris friction, posterior synechiae, and chronic uveitis. *Alters anterior chamber angle structure, narrows angle, and disrupts aqueous humor outflow path, leading to elevated intraocular pressure.	Practical for complex ocular conditions (e.g., posterior capsule complications during cataract surgery, difficult capsular bag opening in secondary implantation).	*Significantly increased risk of secondary glaucoma. *Prone to anterior chamber angle narrowing and aqueous humor outflow obstruction. *Mechanism of inducing secondary glaucoma is not fully clear.	(2, 23, 24)
Medication factor corticosteroids	Induces elevated intraocular pressure (higher susceptibility in children than adults)	*Corticosteroids may disrupt trabecular meshwork function, reducing aqueous humor outflow. *Infants/children have faster and more sensitive intraocular pressure elevation response to corticosteroids (lower dosage required).	Effectively controls postoperative intraocular inflammatory reactions, which are closely associated with GRAE occurrence.	*Risk of inducing elevated intraocular pressure; higher sensitivity in children increases GRAE risk. *Requires strict control of usage timing and dosage to balance anti-inflammatory effects and pressure elevation risk.	(22, 26, 28–30)
Medication factor intraocular pressure-lowering drugs	No direct GRAE risk (therapeutic role), but improper use may increase indirect risk.	Incomplete safety data in children may lead to ineffective intraocular pressure control or adverse reactions, failing to prevent GRAE progression.	Primary treatment for postoperative elevated intraocular pressure; appropriate selection reduces GRAE severity.	Incomplete safety data in infants/children; improper selection may cause adverse reactions or inadequate pressure control, increasing GRAE-related visual damage risk.	(31, 32)

GRAE, Glaucoma-Related Adverse Events; IOL, Intraocular Lens.

## 4 How to balance the contradictions among surgical methods, surgical timing, and medication use?

Given the relatively high incidence of GRAE after congenital cataract surgery and the potential for irreversible damage to visual function, patients who have received primary or secondary IOL implantation need to closely monitor the intraocular pressure of the surgical eye, the anterior segment structure, the retinal nerve fibers of the fundus, and the optic disc cupping during the visual rehabilitation process.

Some studies suggest that during primary simple cataract extraction if circular posterior capsulotomy and anterior vitrectomy have been performed, peripheral iridectomy can be carried out simultaneously to prevent aqueous humor outflow obstruction and angle closure (31), which facilitates the management of various complex scenarios of subsequent elevated intraocular pressure. Before secondary IOL implantation, clinicians should emphasize the assessment of intraocular pressure and anterior segment, especially the structure of the anterior chamber angle and the situation of the posterior chamber space. Some studies have found that for children with postoperative secondary glaucoma, performing anterior chamber angle synechiolysis alone or in combination with anterior chamber angle incision during secondary IOL implantation can achieve good intraocular pressure-lowering effects (32).

After the inflammation is effectively controlled, clinicians should promptly discontinue corticosteroids or switch to non-steroidal anti-inflammatory drug preparations and continuously monitor the intraocular pressure closely to avoid damage to visual function caused by elevated intraocular pressure. Once an increase in intraocular pressure is detected, a detailed assessment should be carried out to clarify the cause and targeted treatment should be implemented. For transient elevated intraocular pressure, drug treatment is the first choice to lower the intraocular pressure, and drugs with fewer adverse reactions in children, such as prostaglandin analogs and carbonic anhydrase inhibitors, should be selected as much as possible (33).

For refractory elevated intraocular pressure, traditional trabeculectomy has a poor effect, and the European Glaucoma Society recommends implanting an aqueous humor drainage valve (31). Beyond these established approaches, managing pediatric secondary glaucoma—including that secondary to congenital cataract surgery—remains clinically challenging: recent literature indicates that such cases often require multiple surgical interventions to achieve adequate intraocular pressure control, highlighting the need for more tolerable treatment options (34) In this context, Minimally Invasive Glaucoma Surgery (MIGS), such as trabeculotomy, has emerged as a potentially safer alternative, favored for its minimal invasiveness, high safety profile, and faster postoperative recovery (35, 36). By either enhancing aqueous humor outflow through the trabecular meshwork or establishing new drainage pathways, MIGS addresses the core pathophysiology of elevated intraocular pressure while mitigating the risks associated with more invasive procedures. Supporting this, several studies have confirmed that compared with traditional glaucoma surgeries, MIGS reduces the incidence of postoperative complications, making it particularly suitable for pediatric patients where minimizing surgical trauma and preserving ocular structural integrity are paramount (37, 38). While a universal consensus on MIGS for pediatric GRAE has not yet been reached, findings from the majority of existing studies suggest that MIGS holds positive implications for children with glaucoma-related adverse events following congenital cataract surgery.

## 5 Conclusion

GRAE after congenital cataract surgery severely threaten pediatric visual recovery, linked to surgical age, methods, and perioperative medications. Younger surgical age (especially ≤6 months) increases GRAE risk, yet there is no consensus on balancing amblyopia prevention and GRAE avoidance. Additionally, the timing and approach of intraocular lens implantation vary and impact GRAE risk, while the timing and dosage of postoperative early corticosteroid use require strict control. Clinically, closely monitor IOP and anterior segment, prioritize pediatric-friendly IOP-lowering drugs, and consider MIGS for refractory cases. This review aims to synthesize perioperative factors influencing postoperative GRAE incidence; given the current lack of consensus on optimal strategies, surgeons must gain a thorough understanding of these mechanisms to make individualized clinical decisions.
